# Antegrade flexible ureteroscopy-assisted percutaneous nephrolithotomy for staghorn calculi: a prospective randomized controlled study

**DOI:** 10.1007/s00240-024-01528-9

**Published:** 2024-02-10

**Authors:** Yuanjiong Qi, Haonan Xing, Shushuai Yang, Zhongsheng Peng, Yue Chen, Shiyong Qi

**Affiliations:** Department of Urology, Tianjin Institute of Urology, The Second Hospital of Tianjin Medical University, Tianjin, 300211 China

**Keywords:** Percutaneous nephrolithotomy, Antegrade flexible ureteroscopy, Staghorn calculi, Stone branch number, Randomized controlled study

## Abstract

The aim is to compare the efficacy and safety between single percutaneous nephrolithotomy (sPNL) and antegrade flexible ureteroscopy-assisted percutaneous nephrolithotomy (aPNL) for the treatment of staghorn calculi. A prospective randomized controlled study was conducted at the Second Hospital of Tianjin Medical University. A total of 160 eligible patients were included, with 81 in the sPNL group and 79 in the aPNL group. The study first compared the overall differences between sPNL and aPNL. Then, the patients were divided into two subgroups: Group 1 (with less than 5 stone branches) and Group 2 (with 5 or more stone branches), and the differences between the two subgroups were further analyzed. The results showed that aPNL had a higher stone-free rate (SFR) and required fewer percutaneous tracts, with a shorter operation time compared to sPNL (*P* < 0.05). Moreover, aPNL significantly reduced the need for staged surgery, particularly in patients with 5 or more stone branches. Moreover, there were no significant differences in the changes of hemoglobin levels and the need for blood transfusions between the sPNL and aPNL groups, and the incidence of multiple tracts was lower in the aPNL group. The two groups showed comparable rates of perioperative complications. We concluded that aPNL resulted in a higher SFR for staghorn calculi, and required fewer multiple percutaneous tracts, reduced the need for staged surgery, and had a shorter operative time than PNL alone, especially for patients with 5 or more stone branches. Furthermore, aPNL did not increase the incidence of surgical complications.

**Supplementary Information:**

The online version contains supplementary material available at 10.1007/s00240-024-01528-9.

## Introduction

Urolithiasis is a common urological condition worldwide. With the advancements in lithotripsy technology and endourological equipment, various treatment options are available depending on individual patient factors. The primary treatment methods for removing renal calculi include extracorporeal shock wave lithotripsy (SWL), percutaneous nephrolithotomy (PNL), and ureteroscopy (URS) [1]. Due to its safety and effectiveness, PNL has become a standard procedure for treating renal stones > 2 cm [1–3]. The reported stone-free rate (SFR) for PNL is around 90% [4]. However, when mentioning staghorn calculi, the SFR is generally unsatisfactory, and it is challenging to remove all stones through a single percutaneous renal tract [5–7]. Patients may require staged surgery and bear the risk of multiple operations and anesthesia. To achieve better stone removal in a single surgery, the surgeon may need to create multiple percutaneous renal tracts, particularly for stones in multiple or parallel calyces. However, the conventional point is that multiple tracts requires specialists with skilled puncture technique and experience in performing accurate percutaneous puncture, otherwise it may lead to greater bleeding and higher complication rates than the single-tract method [8–10].

With the development of endoscopic surgical technology, endoscopic combined intrarenal surgery (ECIRS) has been introduced as an improvement to eliminate the need for multiple tracts while simultaneously improving the SFR in a single surgery. It has been proven safe and effective for treating large stones, facilitating percutaneous tract, and improving SFR [11]. Several developments have been made in the ECIRS position, including the prone split-leg position (PSL), supine lithotomy position, and Galdakao-Modified Supine Valdivia position (GMSV). In recent years, the ECIRS technique has become widely accepted in clinical practice and is often discussed in clinical research for the staghorn calculi management [12]. However, there are still some concerns that prevent ECIRS from gaining more widespread adoption, because this procedure requires two sets of surgical equipment, namely lithotripsy and endoscopic equipment, and two experienced surgeons. Furthermore, the attendant increased costs are constantly mentioned [12–14]. There is still no clear indication for ECIRS in the treatment of staghorn calculi. With less equipment and surgeons, the PNL combined with anterograde flexible endoscopy technique can also manage staghorn calculi like ECIRS. Pastore et al. demonstrated that the combined laparoscopic and antegrade flexible cystoscope for management of staghorn calculi was feasible and repeatable [15]. PNL combined with flexible nephroscope has also been reported to improve the SFR for staghorn stones [16, 17]. Compared with flexible cystoscope, flexible ureteroscope has a larger bending angle at the front end and a wider observation range. In recent years, several studies have been carried out on antegrade flexible ureteroscopy-assisted PNL (aPNL) for the treatment of staghorn calculi, but all of them were retrospective studies with small samples or case reports [18, 19]. The existing constraints on reusable flexible ureteroscope assisted PNL include a high initial purchase cost, vulnerabilities, high expenditures for repair, and a risk of cross-infection [20]. However, there have been encouraging reports on the improvement of single-use flexible ureteroscope over the past few years, which could solve these problems. Therefore, we conducted a prospective randomized controlled trial to compare the efficacy and safety of single PNL (sPNL) and aPNL in treating staghorn calculi by one urologist as a supplement to ECIRS.

## Materials and methods

### Model experiment in vitro

To investigate the feasibility of aPNL, we first utilized 20 porcine kidneys to establish 20 F percutaneous renal tracts through the dorsal mid calyces. Next, we used flexible cystoscope and flexible ureteroscope to visualize the upper, middle, and lower calyces of the kidney, respectively, and compared the proportion of these calyces that could be visualized by cystoscope or ureteroscope. The experimental procedures are presented in the accompanying video (Supplementary Video S1). In addition, we modeled the kidney in equal proportions to the normal kidney structure. Subsequently, the renal calyces were observed by flexible ureteroscope and flexible cystoscope, respectively (Supplementary Video S2). The results of model experiment were used to assess potential strengths and efficacy of flexible ureteroscope.

### Study design and population

To test the efficacy of sPNL and aPNL procedures, a single-center prospective randomized controlled study was conducted in the Second Hospital of Tianjin Medical University. This trial has been registered at www.chictr.org.cn as ChiCTR2300073822. All surgery procedures were performed by an experienced surgeon with over 500 retrograde intrarenal surgery (RIRS) and PNL cases. The study was approved by the Institutional Ethics Committee of the Second Hospital of Tianjin Medical University (IEC: 43/2020), and informed consent was obtained from each patient upon admission. The study was conducted in accordance with the Declaration of Helsinki.

The primary endpoint was the SFR, and the secondary endpoints included staged surgery rate, operative time, percutaneous tract number, hemoglobin drop, postoperative hospitalization, and postoperative complication. The sample size was determined based on our historical data of the SFR after sPNL and aPNL for staghorn stone (45.7% and 68.3%, respectively). The sample size of 72 per group was calculated using PASS software, with 80% power (1 − *β*) and a type-1 error (*α*) of 0.05. However, the sample size was increased to 80 per group to account for a 10% dropout rate.

From October 2020 to October 2022, a total of 160 patients were recruited at the hospital for the clinical trial. Inclusion criteria were as follows: (1) aged 18-year-old or above; (2) presence of staghorn stone confirmed by non-contrast computed tomography (CT) examination; (3) American Society of Anesthesiology scores of 1–2. Exclusion criteria were as follows: (1) patients with solitary kidney, congenital anomalies (such as horseshoe kidney and ectopic kidney); (2) patients with severe preoperative pyelonephritis; (3) patients with intention towards a specific treatment approach. Eligible patients were randomly assigned to the sPNL or aPNL group with a 1:1 ratio using a computer-generated random table. Preoperatively, all patients underwent urine culture and sensitivity. Patients with urinary tract infections were treated with culture-specific antibiotics until the repeat urine culture was negative, and patients with negative urine cultures received second-generation intravenous cephalosporins during anesthesia.

### Surgical procedure

More detailed routine surgical steps were described in our previous reports [21, 22]. In the present study, all procedures were performed under general anesthesia. After retrograde insertion of a 5 F ureteral catheter into the renal pelvis, the patient was changed to a prone position. Then, a 17.5-gauge coaxial needle (uroVision GmbH) was inserted into the targeted calyx with the guidance of ultrasound. The tract was dilated stepwise up to 18 F or 24 F using a serial fascial dilator (uroVision GmbH) and a same-sized peel-away sheath was then placed. Stone fragmentation and clearance were performed using pneumatic, holmium: YAG laser, ultrasonic, or combined lithotripsy equipment under the supervision of an 8/9.8 F rigid ureteroscope or 20.8 F nephroscope (Richard Wolf).

**Fig. 1:**
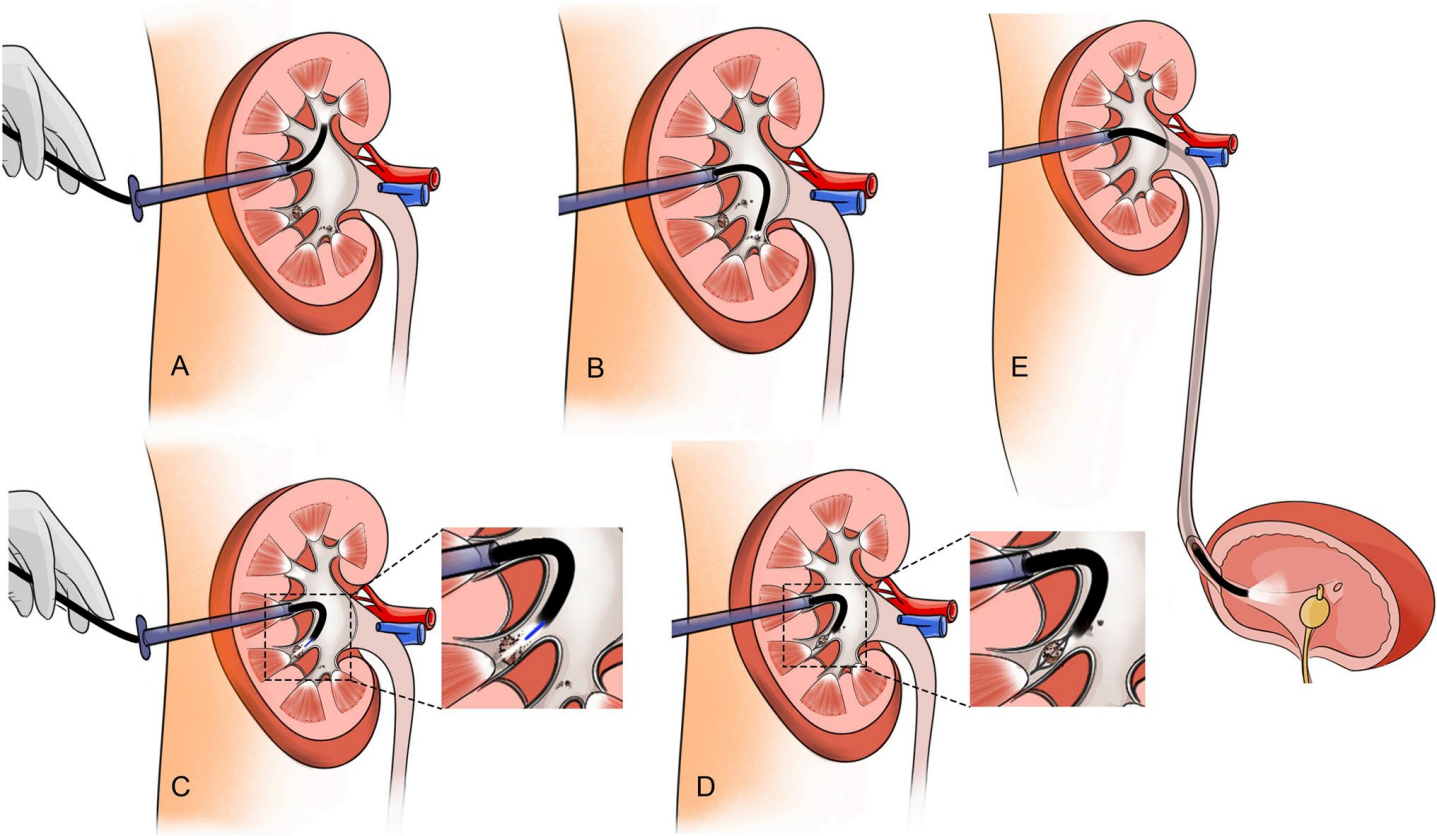
Flexible ureteroscopy and management of residual stones using Tianjin Institute of Urology (TJIU) technique at the end of the lithotripsy procedure: **A** Flexible ureteroscopy of upper calyx. **B** Flexible ureteroscopy of lower calyx. **C**, **D** Flexible ureteroscopy of parallel calyx and management of residual stones. **E** Flexible ureteroscopy of ureter and bladder

Ultrasound was routinely used to check for residual stones at the later stage of the procedure. For sPNL, additional assisted percutaneous renal tracts were established using a similar method if residual stones could not be reached through the initial percutaneous tract. For aPNL, we firstly attempted to use the flexible ureteroscope (AnQIng® Innovex) through the initial middle tract to disintegrate the residual stones into fragments under direct visualization. The high energy setting of the holmium laser was applied to fragment residual stones as far as possible. The stone fragments were then removed by stone basket or washed out through the sheath by irrigation directly. For the residual stone located in the lower calyx or difficult to be fragmented directly, a stone basket was used to translocate into the renal pelvis, where the rigid ureteroscope could reach. If routine ultrasound during the procedure still revealed residual stones, which neither rigid ureteroscope nor flexible ureteroscope could reach, then the second assisted tract was established to remove the stones. In order to further improve the SFR and avoid missing observation of any calyx, we designed the Tianjin Institute of Urology (TJIU) technique, in which the surgeon observed each calyx in a specific order, namely the upper (Fig. 1A), lower (Fig. 1B), parallel calyx (Fig. 1C, D) and the whole ureter (Fig. 1E) using flexible ureteroscope. When flexible ureteroscopy still revealed residual stones, we would disintegrate the stones through the initial or second tract firstly. If the flexible ureteroscope could not reach the residual stones through the initial or second tract, the third assisted tract would be established. We then used the established tracts to disintegrate the residual stones and repeated the above surgical procedures until no residual stone was identified. Additional assisted tracts would be established if needed.

Finally, in addition to residual stones, we would routinely check for any blood clots or bleeding after lithotripsy. Then, a 6 F double-J stent was placed antegrade into the ureter, and one or more 14 F nephrostomy tubes were inserted in all patients.

The surgical procedures are presented in the accompanying video (Supplementary Video S3).

### Evaluation

Urological CT is typically performed on the second postoperative day to evaluate the SFR (stone-free rate with no residual fragments larger than 4 mm). Operative time is defined as the time from the beginning of the puncture to the completion of the last nephrostomy tube placement. Hemoglobin decrease was calculated as the difference between the preoperative level and the level 24 h postoperatively. Postoperative complications were classified according to the Clavien–Dindo grading system. Fever was defined as a body temperature higher than 38.5 ℃. In addition, patient and stone characteristics, the number of intraoperative percutaneous tracts, and the proportion of staged surgery were recorded for both groups.

### Indication and methods of staged surgery

Patients with residual fragments larger than 4 mm would be suggested to undergo additional procedures to attain stone-free status. In consultation with the patient’s preferences, the staged surgery methods consisted of PNL (combined with antegrade flexible ureteroscopy when required) using the same percutaneous renal tract in the primary surgery, retrograde intrarenal surgery (RIRS), or SWL, determined by the stone size and location.

### Subgroup analysis

Our previous study [21] demonstrated that when the number of stone branches was five or more, there was an increased probability of multiple percutaneous tracts, staged surgery, and longer postoperative hospital stays, while the stone-free rate (SFR) decreased. Therefore, we divided patients into Group 1 (stone branch number < 5) and Group 2 (stone branch number ≥ 5) and further analyzed the differences between the two subgroups.

### Statistical analysis

Statistical analysis was conducted using R × 64 4.1.2 statistical software. Continuous variables are presented as mean ± standard deviation (SD), while categorical variables are presented as numbers or percentages. Student’s *t*-test, Chi-square tests, and Fisher’s exact tests were used as necessary. *P* value less than 0.05 was considered statistically significant.

## Results

### Outcome of model experiment in vitro

Compared with flexible cystoscope, flexible ureteroscope had a smaller diameter but a larger bending angle of the anterior end. In addition, the results of model experiment indicated that the proportion of upper, middle, or lower calyx visible with flexible ureteroscope is higher than that with cystoscope. There was a statistically significant difference between flexible ureteroscope and cystoscope in terms of observing the middle calyx and the ability to simultaneously visualize the upper, middle, and lower calyx (*P* < 0.05, Table S1). In addition, model experiment in vitro proved that the flexible ureteroscope allowed for clear visualization of most the renal calyces and the ureter, while the flexible cystoscope did not provide such clear visibility, especially for the parallel renal calyces and certain parts of the upper and lower renal calyces. We also found that the passive bending ability of flexible ureteroscope was better than flexible cystoscope (Supplementary Video S2).

### Study population and baseline characteristic

**Fig. 2:**
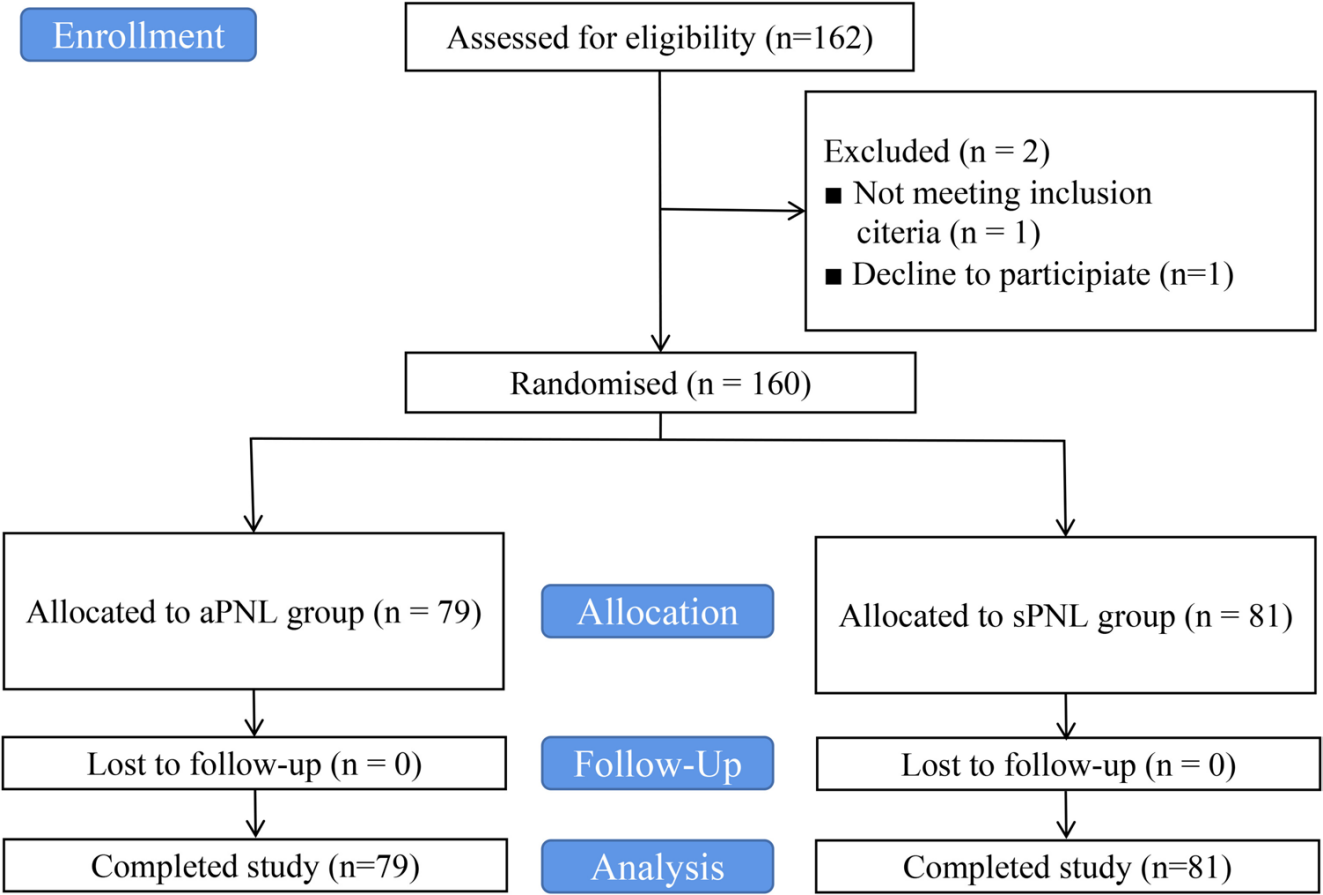
Flow diagram of the present study. *aPNL* antegrade flexible ureteroscopy-assisted percutaneous nephrolithotomy, *sPNL* single percutaneous nephrolithotomy

After excluding patients who did not meet the inclusion criteria or declined to participate, 160 patients were randomly assigned to the two study groups (81 in the sPNL group and 79 in the aPNL group; Fig. 2). There was no significant difference between the groups in terms of age, gender, body mass index (BMI), basic diseases, or laterality. Other preoperative clinical characteristics, including stone burden, stone composition, and hydronephrosis grade, were also comparable (Table 1).

**Table 1:** Baseline characteristic

	sPNL	aPNL	*P*
Number of patients	81	79	
Age, years (mean ± SD)	55.12 ± 11.82	54.03 ± 11.38	0.550
Gender, *N* (%)			
Male	52 (64.20)	58 (73.42)	0.277
Female	29 (35.80)	21 (26.58)	
BMI, kg/m^2^ (mean ± SD)	26.06 ± 3.61	26.50 ± 3.40	0.425
Hypertension, *N* (%)	34 (41.98)	29 (36.71)	0.603
Diabetes, *N* (%)	12 (14.81)	18 (22.78)	0.276
Cardiovascular disease, *N* (%)	7 (8.64)	5 (6.33)	0.799
Pre-WBC, μmol (mean ± SD)	6.51 ± 1.58	6.75 ± 1.91	0.385
Initial positive urine culture, *N* (%)	21 (25.93)	17 (21.52)	0.639
Laterality, *N* (%)			
Left	56 (69.14)	51 (64.56)	0.655
Right	25 (30.86)	28 (35.44)	
Stone burden, cm (mean ± SD)	4.59 ± 2.26	4.16 ± 1.67	0.178
Stone branch number, *N* (%)			
< 5	32 (39.5)	32 (40.5)	1.000
≥ 5	49 (60.5)	47 (59.5)	
CT value of stone, HU (mean ± SD)	991.56 ± 280.32	992.33 ± 316.01	0.987
Main stone composition, *N* (%)			
Calcium oxalate	60 (74.07)	61 (77.22)	0.672
Uric acid	4 (4.94)	5 (6.33)	
Carbonate apatite	12 (14.81)	8 (10.13)	
Ammonium magnesium phosphate	1 (1.24)	3 (3.79)	
Others	4 (4.94)	2 (2.53)	
Hydronephrosis grade, *N* (%)			
No	13 (16.05)	10 (12.66)	0.599
Mild	47 (58.02)	46 (58.23)	
Moderate	13 (16.05)	18 (22.78)	
Severe	8 (9.88)	5 (6.33)	

*BMI* body mass index, *WBC* white blood cell, *sPNL* single percutaneous nephrolithotomy, *aPNL* antegrade flexible ureteroscopy-assisted percutaneous nephrolithotomy

### Efficacy of antegrade flexible ureteroscopy-assisted PNL method

Overall surgery parameters and clinical outcome are summarized in Table 2. The mean surgery time, between the beginning of the puncture and the completion of the last nephrostomy tube placement, for aPNL and sPNL were 77.75 and 83.09 min, respectively. aPNL group yielded shorter operative times but achieved a better stone clearance rate (70.89% vs 51.85%) than sPNL group (*P* < 0.05). The pictures of flexible ureteroscope and cystoscope are shown in Fig. S1.

**Table 2:** Overall surgery parameters and clinical outcome

	sPNL (*n* = 81)	aPNL (*n* = 79)	*P*
Stone free rate, *N* (%)	45 (51.85)	56 (70.89)	0.021
Operative time, min (mean ± SD)	83.09 ± 19.00	77.75 ± 12.94	0.039
Percutaneous tract, *N* (%)			
Single	62 (76.54)	72 (91.14)	0.022
Multiple	19 (23.46)	7 (8.86)	
First sheath size, *N* (%)			
18F	65 (80.25)	68 (86.08)	0.439
24F	16 (19.75)	11 (13.92)	
Hemoglobin drop, g/l (mean ± SD)	1.73 ± 9.04	0.32 ± 7.39	0.281
Staged surgery, *N* (%)	26 (32.10)	12 (15.19)	0.020
Fever (≥ 38.5 ℃), *N* (%)	5 (6.17)	9 (11.39)	0.374
Required blood transfusion, *N* (%)	3 (3.70)	0	0.245
Septic shock, *N* (%)	0	0	–
Postoperative hospitalization, d (mean ± SD)	5.46 ± 1.43	5.38 ± 1.07	0.700

*sPNL* single percutaneous nephrolithotomy, *aPNL* antegrade flexible ureteroscopy-assisted percutaneous nephrolithotomy

### Safety of antegrade flexible ureteroscopy-assisted PNL method

As listed in Table 2, our study found that multiple percutaneous tracts were used more frequently in the sPNL group than the aPNL group (23.46% vs 8.86%, *P* = 0.022). The sPNL group had a higher hemoglobin drop and transfusion rate than the aPNL group, but there were no significant differences between the groups (*P* > 0.05). The hemoglobin drop was calculated as the postoperative hemoglobin value minus the preoperative hemoglobin value, and the standard deviation was greater than the mean due to some patients having elevated postoperative hemoglobin due to blood concentration. Regarding postoperative fever, septic shock, staged surgery and postoperative hospitalization, there was no difference between the groups.

According to the Clavien–Dindo grade system (Table 3), there was no statistically significant difference in complication rates between the two groups. Grade I complications, which include postoperative pain and fever treated with antipyretic therapy, accounted for most complications in both groups (93.83% in sPNL and 98.73% in aPNL). The high proportion of grade I complications may be due to the use of non-steroidal anti-inflammatory drugs for pain relief, which is consistent with enhanced recovery after surgery. In the sPNL group, grade II complications (requiring blood transfusion or additional antibiotics) were seen in 4.93% of patients, and grade III complications (requiring arterial embolization) were seen in 1.23% of patients. In contrast, no patients in the aPNL group had a Clavien grading score between II and IV. However, one patient in the aPNL group experienced a grade V complication (postoperative death) due to pulmonary embolism. Overall, the aPNL group had a lower rate of high-grade complications (1.27% vs 6.16%).

**Table 3:** Perioperative complication based on Clavien–Dindo grade system

Clavien grade, *N* (%)	sPNL (*n* = 81)	aPNL (*n* = 79)	*P*
Grade 1			0.104
Postoperative pain	72 (88.89)	71 (89.87)	
Fever with antipyretic therapy	4 (4.94)	7 (8.86)	
Grade 2			
Blood transfusion	3 (3.70)	0	
Infection requiring additional antibiotics	1 (1.23)	0	
Grade 3			
A (Embolization)	1 (1.23)	0	
B	0	0	
Grade 4			
A	0	0	
B	0	0	
Grade 5			
Death (Pulmonary embolism)	0	1 (1.27)	

*sPNL* single percutaneous nephrolithotomy, *aPNL* antegrade flexible ureteroscopy-assisted percutaneous nephrolithotomy

### Impact of stone branch number on surgical parameters and clinical outcome

Based on our previous research [21], we divided the patients into Group 1 (stone branch number < 5) and Group 2 (stone branch number ≥ 5), and further performed subgroup analysis. Table 4 presents the results of the subgroup analysis. In Group 2, aPNL demonstrated a higher SFR (70.2% vs 46.9%), fewer multiple percutaneous renal tracts (12.8% vs 32.7%), and a lower staged surgery rate (17.0% vs 38.8%) with shorter operative times compared to sPNL (*P* < 0.05). However, in Group 1, these surgical parameters and clinical outcomes were similar between aPNL and sPNL.

**Table 4:** Surgery parameters and clinical outcome of the subgroups

	Group 1			Group 2		
	sPNL	aPNL	*P*	sPNL	aPNL	*P*
	(*n* = 32)	(*n* = 32)		(*n* = 49)	(*n* = 47)	
Stone free rate, *N* (%)	19 (59.4)	23 (71.9)	0.430	23 (46.9)	33 (70.2)	0.035
Operative time, min (mean ± SD)	72.06 ± 10.19	69.88 ± 8.05	0.345	90.29 ± 20.02	83.11 ± 12.95	0.039
Multiple percutaneous tract, *N* (%)						
Single	29 (90.6)	31 (96.9)	0.613	33 (67.3)	41 (87.2)	0.038
Multiple	3 (9.4)	1 (3.1)		16 (32.7)	6 (12.8)	
First sheath size, *N* (%)						
18F	26 (81.2)	28 (87.5)	0.731	39 (79.6)	40 (85.1)	0.660
24F	6 (18.8)	4 (12.5)		10 (20.4)	7 (14.9)	
Staged surgery, *N* (%)	7 (21.9)	4 (12.5)	0.509	19 (38.8)	8 (17.0)	0.032
Postoperative hospitalization, d (mean ± SD)	5.97 ± 2.01	5.31 ± 0.90	0.099	5.12 ± 0.73	5.43 ± 1.17	0.134

*Group 1* stone branch number < 5, *Group 2* stone branch number ≥ 5, *sPNL* single percutaneous nephrolithotomy, *aPNL* antegrade flexible ureteroscopy-assisted percutaneous nephrolithotomy

## Discussion

The European and American Urological Association Guidelines recommend percutaneous nephrolithotomy (PNL) as the first-line treatment for large and complex kidney stones [1, 23]. However, using a single tract for PNL in staghorn calculi has always been challenging due to the higher likelihood of residual stones, despite advances in technology and instrumentation [12].

To achieve an excellent SFR during treatment, several strategies have been proposed in clinical practice. Large et al. [24] demonstrated that secondary procedures were required in 39% of patients treated with single-access PNL for staghorn renal calculi. This process increased the number of anesthesia sessions, costs, and patient discomfort. The location, staghorn status, and size of stones are important factors in determining the percutaneous tract required. Under certain circumstances, multiple percutaneous tracts or increasing the swing amplitude of the rigid ureteroscope may be necessary during PNL. However, these additional tracts and increased swing amplitude can lead to a higher incidence of complications and postoperative discomfort [10, 25, 26]. Wang et al. [9] and Huang et al. [10] reported that patients treated with additional tracts had a higher incidence of comorbidity and a negative impact on renal function without an increase in SFR.

Endoscopic combined intrarenal surgery (ECIRS) was first reported in 2008 [27]. With the introduction of disposable flexible ureteroscopes and lithotripsy equipment, ECIRS has become a revolutionary approach for treating kidney stones due to its higher one-step SFR and lower morbidity compared to PNL alone [12, 28]. Hamamoto et al. [29] evaluated the ECIRS technique in the prone split-leg (PSL) position for the treatment of staghorn calculi and found it to be a safe and effective approach without increasing the number of percutaneous tracts. In addition, Zhao et al. [30] compared the efficacy and safety of ECIRS in the Galdakao-modified supine Valdivia (GMSV) position to PNL and found that ECIRS in the GMSV position had a significantly higher SFR but lower procedure-associated morbidity than the PNL group. This technique combines the advantages of the high efficacy of PNL and the wide exploration angle of flexible ureteroscope. It can be used simultaneously for ureteroscopy and PNL, reduces postural changes after anesthesia, avoids chest and abdomen compression in the prone position, and facilitates intraoperative anesthesia monitoring. Despite the numerous benefits of ECIRS technique, it requires two groups of surgeons and two sets of imaging displays and lithotripsy equipment. In addition, a high degree of cooperation between surgeons is also required to reduce operation time. There is no clear indication for ECIRS in the treatment of staghorn calculi, so how to select patients who are eligible for ECIRS before surgery is a problem that needs to be solved urgently.

Therefore, PNL needs further improvement for higher efficacy, fewer complications, and lower costs. Prior to the application of the ECIRS technique, anterograde flexible cystoscopy or flexible nephroscopy which allowed for the use of thicker laser fibers was performed to improve the SFR of staghorn stones. This surgical approach offers higher lithotripsy efficiency, faster flushing and clearer vision [15–17]. However, the caliber of the cystoscope and nephroscope makes it difficult to perform anterograde examination of the ureter, which increases the risk of larger stones remaining in the ureter. Moreover, our model experiments in vitro demonstrated that flexible ureteroscope was superior to flexible cystoscope in the observation of various renal calyces, especially the parallel calyces. That is because flexible ureteroscope has larger degree of distal deflection but a smaller distal outer diameter compared to flexible cystoscope. In the past, reusable flexible ureteroscope has been limited by high initial purchase cost, vulnerabilities, and high expenditures for repair. However, the currently used disposable flexible ureteroscope can reduce the risk of cross-infection and equipment maintenance costs, which makes antegrade flexible ureteroscopy-assisted percutaneous nephrolithotomy possible.

Previous studies have suggested that PNL combined with anterograde flexible ureteroscope is a preferred treatment for staghorn calculi, but they were retrospective studies or case reports [18, 19]. Therefore, we conducted the prospective randomized controlled trial to thoroughly investigate the efficiency and safety of aPNL. In our previous research, we found that surgery parameters and clinical outcomes were associated with the number of stone branches. In addition, we observed that staghorn calculi with a stone branch number of five or more were more likely to require multiple tracts and staged PNL, and had lower SFR [21]. The present study data demonstrated that aPNL could improve SFR and reduce the percutaneous tract number with shorter operative time (*P* < 0.05). The shorter operative time in aPNL might be attributed to the reduced percutaneous tract number. Percutaneous tract was established under ultrasound guidance in our center. After the first tract was established, some patients might experience urine extravasation, which could lower the quality of the image and thus increase the operative time. In addition, in order to access the stones, we sometimes had to increase the swing amplitude of the rigid ureteroscope in sPNL group. This procedure might cause bleeding of the percutaneous tract, subsequently blurring the vision during lithotripsy and prolonging the operative time. aPNL could also reduce the need for staged surgeries, particularly for patients with stone branch numbers of 5 or more. However, there was no statistically significant difference in hemoglobin change and blood transfusion rate despite an increase in the number of tracts in this study. The other perioperative complications between the two groups were also comparable.

A flexible ureteroscope can be used to displace stones that cannot be reached by a rigid ureteroscope into the renal pelvis for treatment, or it can directly break them down. The front end of the disposable ureteral flexible ureteroscope has a larger bending angle, allowing it to assist in the examination and treatment of stones that are not visible by a rigid ureteroscope. This is particularly useful for stones located in the parallel calyces of the puncture channel, which can be broken down by Holmium laser lithotripsy under the flexible ureteroscope, or under the rigid ureteroscope after the stone basket has been displaced to the renal pelvis. This one-stage procedure improves the SFR and shortens the operation time. Using this technique limits the swing amplitude of the rigid ureteroscope and reduces the number and size of tracts required during the procedure. This helps to decrease the risk of parenchymal laceration and increase the overall safety of the operation. In addition, performing anterograde flexible ureteroscopy can maintain low-pressure perfusion of the kidney, which helps to decrease the risk of postoperative infection. After the lithotripsy is completed, the flexible ureteroscope can be used to check and manage any residual stones in the ureter.

The current study has some limitations. First, the present surgical approach cannot completely replace other surgical methods. Second, our findings in the present study were based on a relatively small sample at a single center and further validation in a multicenter study with a large size sample is required. Lastly, the length of the flexible ureteroscope used in the study might influence the surgeon’s lithotripsy experience. To address this limitation, a flexible ureteroscope with a more suitable length should be developed in the future.

## Conclusion

aPNL is a promising treatment option for multiple kidney and staghorn stones. It has been found to have a higher SFR and a lower rate of multiple percutaneous tracts, staged surgery, and operative time compared to PNL alone. This is particularly true for patients with a stone branch number of 5 or more. Importantly, aPNL does not increase the incidence of surgical complications.

## Supplementary Information

Below is the link to the electronic supplementary material.

Fig. S1 Flexible ureteroscope and cystoscope

Supplementary file2 (DOCX 13 KB)

Supplementary file3 (MP4 84743 KB)

Supplementary file4 (MP4 45166 KB)

Supplementary file2=5 (MP4 160882 KB)

## Data Availability

The datasets generated and analyzed during the current study are available from the corresponding author on reasonable request.
