# Supplementary figures and images for: Antegrade flexible ureteroscopy-assisted percutaneous nephrolithotomy for staghorn calculi: a prospective randomized controlled study
Source: Urolithiasis. 2024 Feb 10;52(1):33. doi: 10.1007/s00240-024-01528-9 (PMC10858820; doi:10.1007/s00240-024-01528-9)

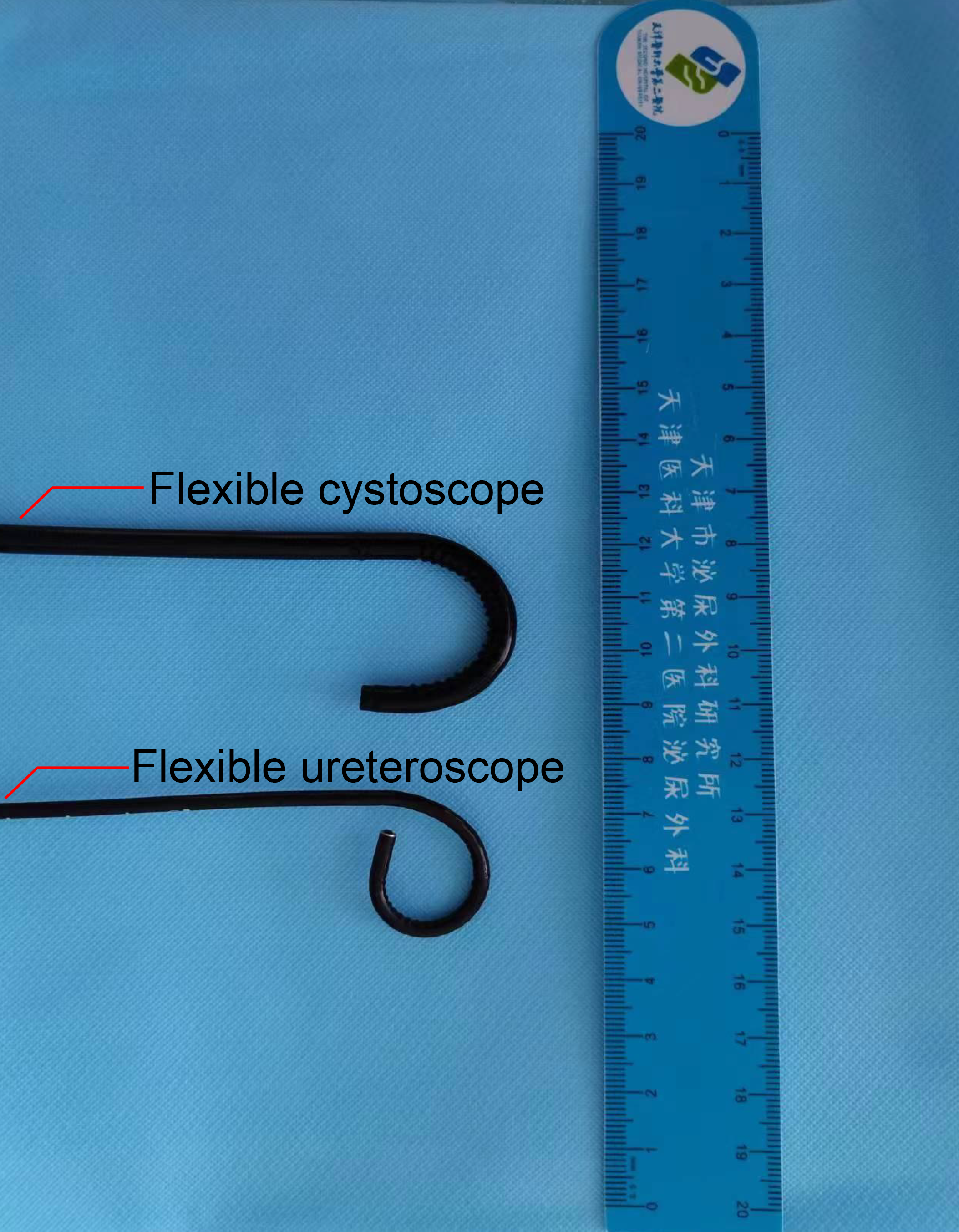

Supplement: Supplementary file 1 — Fig. S1 Flexible ureteroscope and cystoscope [file 240_2024_1528_MOESM1_ESM.tif]
