# Supplementary material for: Antegrade flexible ureteroscopy-assisted percutaneous nephrolithotomy for staghorn calculi: a prospective randomized controlled study
Source: Urolithiasis. 2024 Feb 10;52(1):33. doi: 10.1007/s00240-024-01528-9 (PMC10858820; doi:10.1007/s00240-024-01528-9)
Supplement: Supplementary file 2 — Supplementary file2 (DOCX 13 KB) [file 240_2024_1528_MOESM2_ESM.docx]

| **Table S1** Model experiment in vitro | | | |
| --- | --- | --- | --- |
| Variable, n (%) | Ureteroscope group (n=20) | Cystoscope group (n=20) | *P* |
| Upper calyx |  |  |  |
| No | 0 | 1 (5) | 1 |
| Yes | 20(100) | 19(95) |  |
| Middle/parallel calyx |  |  |  |
| No | 1(5) | 7(35) | 0.0436 |
| Yes | 19(95) | 13(65) |  |
| Lower calyx |  |  |  |
| No | 0 | 2(90) | 0.4872 |
| Yes | 20(100) | 18(10) |  |
| All visible |  |  |  |
| No | 1(5) | 8(40) | 0.01966 |
| Yes | 19(95) | 12(60) |  |
